# Comparison of laboratory characteristics and clinical prognosis of APL with negative and positive PML-RARα gene

**DOI:** 10.1097/MD.0000000000040671

**Published:** 2024-11-22

**Authors:** Xinran Cao, Diyuan Guo, Bin Zhang

**Affiliations:** a Graduate School, Hebei North University, Zhangjiakou, China; b Department of Nuclear Medicine, The Central Hospital in Panzhihua City, Panzhihua, China; c Clinical laboratory, The First Affiliated Hospital of Hebei North University, Zhangjiakou, China.

**Keywords:** acute promyelocytic leukemia, laboratory characteristics, *PML-RARα* gene

## Abstract

This study analyzes the laboratory characteristics and prognosis of patients between *PML-RARα* negative APL and *PML-RARα* positive APL and compares the differences in order to improve the understanding of this rare APL and guide clinical diagnosis and treatment. A total of 81 patients with newly diagnosed APL based on bone marrow cell morphology were included, with 14 in the PML-RARα gene negative group and 67 in the PML-RARα gene positive group. The sex, age, peripheral blood routine test, coagulation related indicators, bone marrow cell morphology, flow cytometric immunophenotype, abnormal chromosome expression and prognosis of the 2 groups were analyzed and compared. *PML-RARα* gene-negative and *PML-RARα* gene-positive groups were statistically significant in leukocyte count, fibrinogen content, proportion of abnormal promyelocytes, positive rate of Auer bodies, strongly positive peroxidase staining, positive CD13, CD4, CD11b, CD15, CD25 expression and complete response rate during 1 course (*P* < .05). By the end of follow-up in February 2021, the duration of CR in the *PML-RARα* gene-negative group was short (*P* < .05). This study found that the efficacy and prognosis of patients with *PML-RARα* gene negative were worse than those of the positive group, but the correlation between indicators and prognosis needs to be further explored and confirmed in more diverse samples.

## 1. Introduction

Acute promyelocytic leukemia (APL) can be divided into typical and atypical. Typical APL is a special subtype of acute myelocytic leukemia (AML), which is characterized by abnormal promyelocyte malignant hyperplasia. About 90% to 95% of cases have t(15; 17)(q22; q21), and produces the *PML-RARα* fusion gene, whose sensitivity to treatment with all-trans retinoic acid (ATRA) and arsenic trioxide makes it the best curable subtype of AML.^[1]^ PML-RARα fusion gene is a marker of APL. However, it has been reported in recent years^[2]^ that 1 or 2 items of bone marrow cell morphology and flow immunophenotype were similar to APL in some cases, but no *PML-RARα* fusion gene was detected, that is, atypical APL with negative *PML-RARα* gene. Due to the lack of specific manifestations and therapeutic targets, there are certain challenges in diagnosis and treatment. A total of 81 patients with APL (including 14 patients with *PML-RARα* negative and 67 patients with *PML-RARα* positive) were analyzed and compared with peripheral blood routine, coagulation related indexes, bone marrow imaging, flow immunophenotype, abnormal chromosome expression and clinical prognosis. The aim is to improve the understanding of laboratory and clinical staff on this rare type of APL, provide objective and reliable basis for rapid diagnosis, clinical rational drug use and prognosis assessment of patients, and reduce the misdiagnosis rate of this disease at the first diagnosis.

## 2. Materials and methods

### 2.1. Research object

A total of 81 patients admitted to the Department of Hematology of the First Affiliated Hospital of Hebei North University from May 2012 to February 2021 who were initially diagnosed with APL based on bone marrow cell morphology and flow immunophenotype were selected and divided into *PML-RARα* negative group and *PML-RARα* positive group according to molecular biological examination results. In the negative group, there were 14 cases, male: female = 5:9, median age 51 years; There were 67 cases in the positive group (male:female = 29:38, median age 48 years). All patient data were obtained from the inpatient electronic medical record system of the First Affiliated Hospital of Hebei North University, and the follow-up deadline was February 28, 2021.

#### 2.1.1. Inclusion and exclusion criteria

Inclusion criteria: Before drug intervention in clinical patients, peripheral blood routine and coagulation function detection had been perfected. It was in line with the Criteria for Diagnosis and Efficacy of Blood Diseases,^[3]^ and the preliminary diagnosis was APL.

Exclusion criteria: Patients with incomplete data are excluded, such as patients who give up treatment midway due to economic or critical conditions.

#### 2.1.2. Induced remission treatment plan

According to the Chinese Guidelines for the Diagnosis and Treatment of Acute promyelocytic Leukemia (2018 edition)^[4]^ and the NCCN guidelines,^[5]^ when APL is highly suspected, treatment should be carried out according to the guidelines.

### 2.2. Main reagent

Cellpack DCL, Cellpack DFL, Sulfolyser, Lysercell^TM^ WNR, Fluorocell^TM^ WNRS, Fluorocell^TM^ WNR, Lysercell^TM^ WDF, Fluorocell^TM^ WDF, Lysercell^TM^ WPC, Fluorocell^TM^ WPC, Fluorocell^TM^ platelet (PLT), Thromborel S, Actin, Thrombin Reagent, Innovance D-Dimer, Ov Buffer, Reye-Giemsa staining solution, peroxidase (POX) staining solution, BD FACS TM Lysing Solution, phosphate buffer saline, BD IntraSure ^TM^ Kit (California).

### 2.3. Detection methods and steps

#### 2.3.1. Routine peripheral blood test

EDTA-K2 anticoagulant venous blood was detected with Sysmex XN-2000 automatic blood analyzer.

#### 2.3.2. Detection of coagulation related indicators

The sodium citrate anticoagulant venous blood was detected by Sysmex CS-5100 automatic coagulation analyzer.

#### 2.3.3. Morphological examination of bone marrow cells

Raysh-giemsa staining and POX staining: well-prepared dry bone marrow smear specimens were selected for Raysh-Giemsa staining and POX staining respectively. After natural drying, microscopic examination and determination of the degree of staining reaction of POX were carried out, and diagnosis was made according to FAB typing criteria.

#### 2.3.4. Flow cell immune typing

Bone marrow cell antigens were labeled by flow cytometry immunofluorescence. BD FACS CANTO II flow cytometry and BD FACSDiva Software in the computer system were used to classify the cells by double-parameter scatterplot of SSC (side scatter)/CD45. The fluorescence intensity of fluorescein-labeled monoclonal antibodies on the cell membrane or in the cytoplasm was detected to determine the content of cell antigen molecules.^[5]^

#### 2.3.5. Molecular genetic testing

EDTA-K2 and heparin anticoagulant bone marrow fluid were used, respectively, and submitted for examination within 2 hours. The test results were provided by the Chinese Academy of Medical Sciences, the Institute of Hematology of Peking Union Medical College and the Hospital of Hematology.

### 2.4. Statistical methods

SPSS25.0 statistical software was used for analysis. The variables conforming to the normal distribution are expressed as $\bar{X}\pm S$ and compared by the *t* test of 2 independent samples. Continuous variables that did not conform to the normal distribution were represented by P50 (P25, P75), and Mann–Whitney *U* test with 2 independent samples was used. Chi-square (χ^2^) test or Fisher exact test were used to compare the rates between the 2 samples, with α = 0.05 as the test level, and *P* < .05 as the difference was statistically significant.

## 3. Result

### 3.1. Basic patient information

#### 3.1.1. Basic data of 14 patients with *PML-RARα* gene negative

Among the 14 patients with *PML-RARα* gene negative, 11 cases had the same karyotype as normal people, and the remaining 3 cases had other chromosomal abnormalities (as shown in Table 1). In the negative group, 12 of the 14 patients were negative for *PML-RARα* gene, and the other 2 were HOX11 and MLL-AF6 genes, respectively. In the negative group, 2 of the 14 patients died early. During the initial induction therapy, 5 patients achieved complete remission (CR) after receiving DA (Daunorubicin + cytarabine), ATRA + DA or CAG (acclarithromycin + cytarabine + granulocyte colony-stimulating factor) regimen, but relapsed later. The remaining 7 patients received DA, IA (daunorubicin + cytarabine), CAG, ATRA + CAG, ATRA + IA, or ATRA + TA (pirarubicin + cytarabine) regimen after 1 course of chemotherapy, 4 patients achieved CR and 3 patients did not achieve CR.

**Table 1:** Basic data of 14 PML-RARα-negative cases

No.	Sex	Age	Bone marrow promyelocytic ratio (%)	Flow cytometric immunophenotype
1	Female	66	19	CD11b,CD13,CD15,CD33,CD38,CD64,CD117,MPO
2	Female	50	95	CD8,CD11b,CD13,CD19,CD25,CD34
3	Female	59	30.5	CD13,CD33,CD38,CD64,CD117,MPO
4	Male	61	23	CD11b,CD13,CD15,CD33,CD64,CD117,MPO
5	Female	60	16.5	CD9,CD13,CD25,CD33,CD34,CD38,CD71,CD117
6	Female	31	73	CD9,CD15,CD33,CD38,CD64,CD117,HLA-DR
7	Female	16	63	CD9,CD13,CD33,CD38,CD117,MPO
8	Male	77	46	CD13,CD15,CD34,CD38,CD56,CD71,CD117,MPO
9	Male	56	67.5	CD11b,CD13,CD15,CD25,CD33,CD34,CD38,CD56,CD64,CD117, MPO
10	Male	43	20.5	CD33,CD38,CD64,CD117,MPO
11	Male	66	35	CD15,CD33,CD38,CD117,MPO
12	Female	18	67.5	CD4,CD11b,CD13,CD15,CD33,CD38,CD56,CD64,CD117,MPO,HLA -DR
13	Female	58	67	CD15,CD33,CD38,CD56,CD117,MPO
14	Female	50	93	CD4,CD13,CD15,CD33,CD38,CD56,CD64,MPO,HLA-DR
No.	Chromosome karyotype	Gene		First treatment plan and prognosis
1	46,XX[20]	PML-RARα(2013)		ATRA × 10 d, died of cerebral hemorrhage
2	46,XY[19]	PML-RARα(–)		(ATRA + DA) × 7 d didn’t achieve CR, achieved CR after the second course of DA, and relapsed after the third course of DA
3	46,XX[11]	HOX11		DA × 7 d achieved CR, relapsed after 18 months
4	46,XY[4]	PML-RARα(–)		DA × 7 d achieved CR, relapsed after 8 months
5	46,XX[13]	PML-RARα(–)		DA × 7 d achieved CR, abandoned treatment in critical condition after CAG × 14 d
6	46,XX,t(6;11)(q27;q23)[3]/ 46,XX[17]	MLL- AF6		ATRA × 5 d, IA × 7 d achieved CR
7	46,XX[20]	PML-RARα(–)		IA × 7 d didn’t achieve CR
8	46,XY[20]	PML-RARα(–)		DA × 7 d didn’t achieve CR
9	44,X,-Y,del(5)(q13),der(15),16, -21,+mar[20]	PML-RARα(–)		DA × 7 d didn’t achieve CR, died after (decitabine + CAG) × 14 d
10	46,XX[20]	PML-RARα(–)		ATRA × 5 d + TA × 7 d achieved CR
11	46,XY[19]	PML-RARα(–)		CAG × 14 d achieved CR
12	46,XX[16]	PML-RARα(–)		(ATRA + DA) × 7 d achieved CR, relapsed after 8 months
13	46,XX[20]	PML-RARα(–)		ATRA × 16 d + CAG × 7 d achieved CR
14	47,X,(X;10)(p12;p11),+8[11] 48,idem,+21[7]/46,XX[2]	PML-RARα(–)		CAG × 14 d achieved CR, relapsed after 7 months

CAG = ACLA + Ara-C + G-CSF, DA = DNR + Ara-C, IA = IDA + Ara-C, PML-RARα = promyelocytic leukemia-retinoic acid receptor-α, TA = THP + Ara-C.

#### 3.1.2. Basic data of 67 patients with positive *PML-RARα* gene

Among 67 patients with *PML-RARα* gene positive, 11 were in high risk group, 33 were in middle risk group, and 23 were in low risk group.

### 3.2. Comparison of gender, age, peripheral blood routine, coagulation related indexes and bone marrow images between the 2 groups

**Figure 1.**
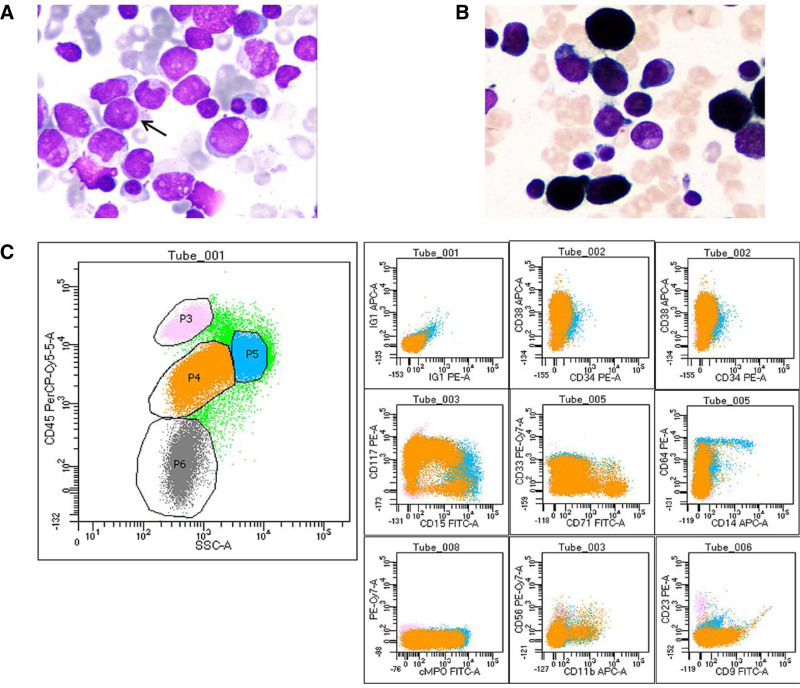
Morphology and immunophenotype of PML-RARα negative. Abnormal promyelocytes (arrows indicated) with varying cell sizes and low cytoplasm content were found in bone marrow smear stained with Raysh-Giemsa (magnification ×1000, panel A). Pseudopod-like processes, purple-red small azurin particles, folded nuclei, clear nucleoli, and no Auer bodies were seen. The staining intensity of POX was strongly positive (panel B). Immunological analysis using flow cytometry was performed on 100,000 cells from the patient’s bone marrow. In the two-dimensional scatterplot of SSC/CD45, 64.1% of the cells were P4 cells, and the fluorescence intensity of CD45 PerCP and SSC were moderately expressed (about 103) (panel C left). The immunophenotype of P4 cells was as follows: CD34-/CD38+, HLA-DR-/CD13+, CD15-/CD117+, CD71-/CD33+, CD14-/CD64-, cMPO+, CD11b-/CD56-, CD9 + (21.8%)/CD23- (panel C). Consistent with myeloid naive cell immunophenotype. Molecular genetic results: 46, XX [20]; PML-RARα (-).

**Figure 2.**
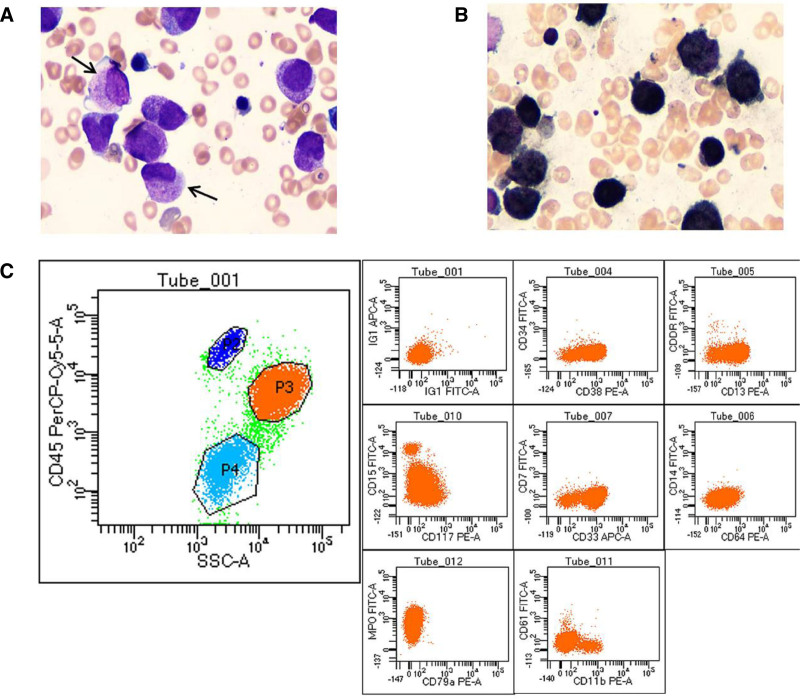
Morphology and immunophenotype of PML-RARα positive. Bone marrow smear stained with Raysh-Giemsa (magnification × 1000, panel A) revealed abnormal promyelocytes (arrows indicated) with varying sizes and irregular appearance, rich cytoplasm content, visible internal and external cytoplasm stratification, more fine and dispersed azophil particles in the inner layer, no or few particles in the outer cytoplasm, propodia protrusion in some cells, round or oval nuclei, thick chromatin, and indistinct nucleolus. Faggot-like Auer bodies were found in some cells. POX staining was strongly positive (panel B). Immunological analysis using flow cytometry was performed on 100,000 cells from the patient’s bone marrow. In the two-dimensional scatterplot of SSC/CD45, the P3 cell group accounted for 73.7%, and the CD45 PerCP showed moderate fluorescence intensity expression, while the SSC fluorescence intensity was relatively strong expression (about 104) (panel C left). The immunophenotype of P3 cell group was as follows: CD38+/CD34-, CD13+/HLA-DR-, CD117+/CD15-, CD33+/CD7-, CD64-/CD14-, cMPO+/cCD79a-, CD11b-/CD61- (panel C). Consistent with myeloid naive cell immunophenotype. Molecular genetic results: 46, XY, t (15; 17)(q22; q21) [15]/46, XY [5]; PML-RARα (+).

There was no significant difference in gender and age between the 2 groups (as shown in Table 2). There were statistically significant differences in white blood cell counts (*P* < .05), but not in hemoglobin (Hb) and PLT counts (*P* > .05). There was significant difference in fibrinogen (FIB) content (*P* < .05), and the thrombin time (prothrombin time), activated partial thromboplastin time and plasma D-dimer had no statistical significance (*P* > .05). The positive rate of abnormal promyelocytes and Auer bodies in bone marrow and the degree of staining reaction of POX were strongly positive had statistical significance (*P* < .05), while the proportion of positive and weakly positive degree of staining reaction in POX had no statistical significance (*P* > .05).The morphological and immunophenotypic characteristics of *PML-RARα* negative are shown in Figure 1, and the morphological and immunophenotypic characteristics of *PML-RARα* positive are shown in Figure 2.

**Table 2:** Comparison of peripheral blood routine, coagulation related indexes and bone marrow cell morphology between PML-RARα negative group and positive group

		PML-RARa negative group (n = 14)	PML-RARa positive group n (n = 67)	*P*
Male/female		5/9	29/38	.602
Age (years)		50.79 ± 18.03	47.94 ± 15.73	.590
Peripheral blood routine test				
WBC (×10^9^·L^−1^)		13.43 (2.76, 52.06)	2.02 (1.25, 5.82)	.005
Hb (g·L^−1^)		80.64 ± 18.58	80.18 ± 12.31	.942
PLT (×10^9^·L^−1^)		43.21 ± 13.34	31 (15, 48)	.532
Coagulation related indicators				
PT (s)		12.74 ± 1.27	13.3 (12.0, 16.3)	.177
APTT (s)		27.18 ± 5.47	26.4 ± 3.89	.676
FIB (g·L^−1^)		2.74 (2.48, 5.02)	1.66 (1.02, 2.95)	.005
DD (mg·L^−1^)		8.16 (2.10, 26.11)	9.95 (3.56, 27.63)	.764
Bone marrow cell morphology				
Bone marrow promyelocytic ratio (%)		53.68 ± 16.20	87.5 ± 12.90	.000
Auer body positive rate (%)		21.43	88.06	.000
POX staining reaction degree	Strong positive (%)	42.86	8.59	.007
	Positive (%)	28.57	1.45	.091
	Weak positive (%)	28.57	8.96	.065
	Negative (%)	0	0	–

APTT = activated partial thromboplastin time, DD = D-dimer, FIB = fibrinogen, Hb = hemoglobin, PLT = platelet, PML-RARα = promyelocytic leukemia-retinoic acid receptor-α, POX = peroxidase, PT = prothrombin time, WBC = white blood cell.

### 3.3. Comparison of flow immunophenotypes between the 2 groups

There were statistically significant differences in the positive expression rates of CD4, CD11b, CD13, CD15, and CD25 between the negative and positive groups of *PML-RARα* gene (*P* < .05), but no statistically significant differences in the positive expression rates of other antigens (*P* > .05) (as shown in Table 3).

**Table 3:** Comparison of flow cytometric immunophenotypes between PML-RARα negative group and positive group

	CD2	CD4	CD7	CD8	CD9	CD11b	CD12	CD13	CD15	CD19
PML-RARaNegative group (n)	0	2	0	1	3	5	0	10	9	1
Positive rate (%)	0.00	14.29	0.00	5.88	21.43	35.71	0.00	71.43	65.29	5.88
PML-RARaPositive group (n)	2	0	1	0	6	8	1	65	26	2
Positive rate (%)	2.99	0.00	1.49	0.00	8.96	11.94	1.49	97.01	38.81	2.99
*P*	1.000	.028	1.000	.173	.183	.043	1.000	.005	.080	.439
	CD25	CD33	CD34	CD38	CD56	CD71	CD64	CD117	MPO	HLA-DR
PML-RARaNegative group (n)	3	12	4	13	5	2	8	12	10	4
Positive rate (%)	21.43	85.71	28.57	92.86	35.71	14.29	57.14	85.71	71.43	28.57
PML-RARaPositive group (n)	0	62	6	60	10	7	42	63	61	7
Positive rate (%)	0.00	92.54	8.96	89.55	14.93	10.45	62.69	94.03	91.04	10.45
*P*	.004	.598	.065	1.000	.122	.650	.698	.276	.065	.091

PML-RARα = promyelocytic leukemia-retinoic acid receptor-α.

### 3.4. Comparison of the detection rate of abnormal chromosomes between the 2 groups

The detection rates of abnormal chromosomes in the negative group and the positive group were 21.43% and 22.39%, respectively (the specific karyotypes of 3 abnormal chromosomes in the negative group and 15 abnormal karyotypes in the positive group were shown in Table 1), and there was no statistical significance between the 2 groups (*P* > .05) (As shown in Tables 4 and 5).

**Table 4:** Comparison of abnormal karyotype detection rate between PML-RARα negative group and positive group

	PML-RARα negative group	PML-RARα positive group	*P*
The detection rates of abnormal karyotypes	21.43%	22.39%	1.000

PML-RARα = promyelocytic leukemia-retinoic acid receptor-α.

**Table 5:** 15 cases of abnormal karyotype in PML-RARα positive group

No.	Chromosome karyotype
1	46,XY,t(15;17)(q22;q21)[7]/47,idem,+21[8]/46,XY[1]
2	46,XY,add(11)(q23),t(15;17)(q22;q21)[2]/46,XY[6]
3	46,XX,der(15)t(15;17)(q22;q21),der(17)add(17)(p12)t(15;17)[20]
4	46,XX,t(15;17)(q22;q21)[16]/47,idem,+mar[2]/46,XX[2]
5	47,XX,+8,der(15)t(15;17)(q22;q21),ider(17)(q10)t(15;17)[13]/47,t(15;17)(q22;q21), +mar[5]/46,XX[2]
6	46,XY,der(15)t(15;17)(q22;q21),ider(17)(q10)t(15;17)[12]/46,XY,t(15;17)(q22;q21) [4]/46,XY[4]
7	46,XX,del(7)(q11q22),t(15;17)(q22;q21)[12]/46,XX
8	46,XX,der(1)(p36)del(1)(q22q24),add(3)(p25),add(4)(q28),del(7)(q32),del(11)(p11), add(16)(q22)[8]/47,idem,+8[12]
9	46,XY,del(11)(p14),t(15;17)(q22;q21)[17]/46,XY[3]
10	46,XX,t(6;21)(q11;q21),t(15;17)(q22;q21)[19]/46,XX[1]
11	47,XX,del(7)(q12q22),t(15;17)(q22;q21),+mar[7]/46,XX[13]
12	45,XY,-6,add(7)(q36),t(15;17)(q22;q21),t(16;18)(q12;p11),add(19)(q12)[9]/46,idem, +mar[11]
13	46,XY,der(15)t(15;17)(q22;q21),ider(17)(q10)t(15;17)[17]/46,XY[3]
14	46,XY,t(12;19;17;15)(q13;q12;q21;q24)[11]/46,XY[9]
	46,XX,der(15)t(15;17),ider(17q)t(15;17)

PML-RARα = promyelocytic leukemia-retinoic acid receptor-α.

### 3.5. Comparison of initial induction therapy and prognosis between the 2 groups

Complete remission (CR), recurrence rate and duration of CR remission after one course of chemotherapy were statistically significant between the 2 groups (*P* < .05), and there was no statistically significant difference in early mortality rate between the 2 groups (*P* > .05) (As shown in Table 6).

**Table 6:** Comparison of treatment and prognosis between PML-RARα negative group and positive group

	PML-RARα negative group	PML-RARα positive group	*P*
n	14	67	
1 course CR rate	57.14%	88.06%	.013
Early mortality	14.29%	1.49%	.076
Relapse rate	35.71%	2.99%	.001
CR duration (d)	39.50 (21, 223)	1031 (683, 2032)	.000

CR = complete remission, PML-RARα = promyelocytic leukemia-retinoic acid receptor-α.

## 4. Discuss

APL is a unique subtype of AML, which has a characteristic chromosome translocation t(15; 17)(q22; q21) and *PML-RARα* fusion gene. By comparing the laboratory characteristics of PML-RARα gene negative group and positive group, we learned that there were no significant differences between the patients of *PML-RARα* gene negative group and positive group in age and sex (*P* > .05), but there were similar or different characteristics in peripheral blood routine, coagulation related indexes and bone marrow imaging.

In terms of routine peripheral blood, myeloid hyperplasia was significantly active in patients with *PML-RARα* gene negative group, while the obvious increase of white blood cells would affect the adhesion and migration of leukemia cells, resulting in increased blood viscosity and increased risk of thrombosis and microcirculation disturbance.^[6]^ From the numerical point of view, both the negative group and the positive group had a significant reduction of Hb and PLT, and the reduction of PLT directly increased the risk of bleeding in patients.

High early mortality of APL is attributable to easy bleeding, diffuse intravascular coagulation complications, due to high coagulation-promoting and fibrinolytic activity of APL cells. As a clotting substance, FIB is involved in coagulation. Data^[7]^ show that the level of FIB is negatively correlated with tumor load, that is, the greater the tumor load of acute leukemia, the more serious the abnormal coagulation function and the higher the risk of bleeding. However, although the patients with *PML-RARα* gene negative group had higher white blood cell and greater tumor load, the content of FIB was also higher, which may be due to the tendency of abnormal coagulation in the 14 patients in this study at the time of initial detection, and the obvious increase of D-dimer also suggested the risk of thrombosis. Continuous hypercoagulability will deplete coagulation factors and PLT, and increase the risk of bleeding. Therefore, dynamic monitoring of blood routine and coagulation function in patients with suspected APL is one of the important measures to reduce early mortality.

The percentage of abnormal promyelocytic granulocyte in bone marrow of *PML-RARα* gene negative group was significantly lower than that of positive group (*P* < .05). The proportion of abnormal promyelocytes is an important reference point in the diagnosis of bone marrow cell morphology. If the proportion increase is not obvious or combined with myelocytosis, APL should be judged with suspicion. In addition, POX staining is the first choice and the most important cytochemical staining method to assist in determining the type of acute leukemia. Under normal circumstances, poorly differentiated granulocytes are negative, and well-differentiated granulocytes are positive to moderately mature granulocytes, and the more mature the cell, the higher the degree of positive reaction. Typical APL is rich in azurin granules in promyelocytes, and the staining reaction of POX is mostly strongly positive. In this study, the proportion of strongly positive staining degree of POX in the negative group was lower than that in the positive group (*P* < .05), which may be caused by the presence of atypical genes or the expression of CD34 in some cells, belonging to the transition stage of differentiation of promyelocytic granulocytes.^[8]^ The indistinguishable morphology may also be one of the reasons why the percentage of myeloid promyelocytes in the negative group was lower than that in the positive group.

Flow immunotyping can provide a basis for differential diagnosis when there are doubts about the morphology. The SSC is too large, and the immunophenotypes of CD33(+), CD13(+), MPO(+), CD117(+), CD34(−), HLA-DR(−), and CD11b(−) are typical manifestations of APL^4^. In 67 patients with *PML-RARα* positive APL, the SSC of the naive cell population was about 10^4^. The SSC of the 14 negative patients was smaller, about 10^3^. When the presence of atypical promyelocytes is considered, fewer intracellular particles make the SSC smaller. In this study, the same combination of immunofluorescent antibodies was used to label bone marrow cells. The experimental results showed that: the positive expression rates of CD4, CD11b, CD15, and CD25 in *PML-RARα* gene negative group were higher than those in *PML-RARα* gene positive group (*P* < .05), but the positive expression rate of CD13 was lower (*P* < .05), and there were no statistically significant differences in the positive expression rates of other antigens between the 2 groups (*P* > .05). CD4 and CD25 were only expressed in negative patients, the positive expression rates were 14.29% (2/14) and 21.43% (3/14), respectively, and the expression intensity was in the weak expression range. CD4 is usually expressed in T lymphocytes, while CD25 is normally expressed in T lymphocytes, B lymphocytes and mononuclear macrophages. As an independent adverse prognostic factor for AML, CD25 is mainly found in AML-M4 and AML-M5 subtypes.^[9,10]^ Although the expression intensity of the above cross-line antigens is weak, the significance of predicting the prognosis of negative patients cannot be ignored. The positive expression rates of myeloid markers CD11b and CD15 in the negative group were 35.75% (5/14) and 65.29% (9/14), respectively, and the expression intensity was concentrated in the range of 60% to 70%. CD11b and CD15 are myeloid maturation antigens, and there is evidence^[11]^ that the absence of CD11b can be an important indication for the differentiation of APL from other AML. Although CD15 is normally highly expressed in the promyelocyte stage,^[12]^ its expression rate in APL is low. CD11b and CD15 had higher positive expression rate and expression intensity in the negative group, which was atypical in the flow immunophenotype of *PML-RARα* negative patients. In addition, CD13 is expressed in cells of different stages of granulocyte development and on monocytes. In APL mainly characterized by promyelocytic hyperplasia, CD13 is mostly expressed positively, while in the negative group, the positive expression rate of CD13 is low, which may be the cause of abnormal antigen expression in some heterogeneous cells, which is also different from typical APL.

The incidence of clonal chromosome addition abnormalities in APL can be as high as 30%, the number change is the most common with + 8,^[13]^ and the structural abnormality is the most common with ider(17)(q10).^[14]^ APL patients are affected by t(15; 17) is considered to have a good prognosis,^[15]^ but the prognostic significance of additional chromosomal abnormalities for APL remains controversial. Among 67 patients with *PML-RARα* gene positive, 52 cases had typical chromosome changes and 15 cases had additional chromosome variants, all of which had varying degrees of number changes and/or structural abnormalities, and autosomal + 8 was the most common number changes.

Among 14 *PML-RARα* negative patients, 3 of them had other chromosomal translocations or additional abnormalities. The patient was initially diagnosed with APL due to the confusion of morphology and immunophenotype, but eventually the common chromosomal translocations and fusion genes of other subtypes of AML were detected. It can be seen that the diagnosis by morphology and immunophenotype alone is not comprehensive, and early accurate identification is the key to reduce misdiagnosis of the patient. In addition, one patient was a chimera of 3 different karyotype cell lines, and not only had rare t(X; 10)(p12; p11), and autosomes + 8 and + 21. It has been reported that isolated + 8 and + 21 AML may have a poor prognosis,^[16]^ but there are also reports that their expression has no effect on prognosis.^[17]^ The remaining 1 patient had simultaneous abnormalities in chromosome number and structure, and one clone involved the increase of autochromosomes 16 and 21 and the deletion of sex chromosome Y, which also combined del(5)(q13), der(15), and + mar. At present, there is no study on the prognosis of + 16, +21 and -Y co-expression, but it has been reported that AML with abnormal karyotypes of autosomal monomers has a poor prognosis, especially for patients with 2 or more abnormal karyotypes of monomers in one clone.^[18]^ Moreover, literature also suggests that AML patients with abnormal structure and number of additional chromosomes may have poor prognosis.^[19]^ Whether it is a simple number change or structural abnormality, or the number and structure of chromosomes are both abnormal, the influence of additional abnormalities on the prognosis needs to be accumulated more abundant clinical data. In addition, the data of 14 patients in the negative group as a whole showed that there was no direct correlation between chromosome karyotype complexity and gene abnormality in the negative group.

Chromosomal banding technology or FISH detection were used for cytogenetic detection of all cases in this study, except for the possibility that the analysis results may be affected by low cell number, few metaphone division phases or chromosome culture failure,^[20]^ and this limited fluorescence quantitative reverse transcription polymerase chain reaction technology can be a good solution.^[21]^ However, there is still a possibility of false negative due to the long detection cycle and complicated procedures resulting in RNA degradation. Currently available next generation sequencing technology can detect less than 1% of mutations,^[22]^ serving as an efficient and powerful tool to accurately locate and analyze genes. In this study, the pathogenic gene could not be further identified by next generation sequencing sequencing technology due to factors such as economy or early death, and more samples need to be explored for recurrent gene mutations.

Due to the lack of ATRA and arsenic trioxide targets as opposed to typical APL in chromosome rearrangement and fusion genes, the outcomes and prognosis of these patients vary. In 14 patients with *PML-RARα* negative gene, the efficacy of induction therapy with AML chemotherapy regimen was mixed. The remission rate, early mortality rate, recurrence rate and CR duration in the first course of treatment were compared between the 2 groups. It can be seen that patients with *PML-RARα* gene negative may have a lower CR rate in the first course of treatment, a significantly higher recurrence rate and a shorter CR duration due to poor response to drug therapy. Considering that the recurrence of the disease may aggravate the blow of immune disorders on the body, cause more serious clotting disorders and cause critical illness, and also make the duration of CR shorter, resulting in shorter survival time. Regardless of the efficacy of any chemotherapy regimen, in cases of suspected APL that have not been verified by chromosomes and genes, effective treatment should be taken early to prevent the occurrence of hemorrhagic diseases.

In short, the exploration of laboratory characteristics of *PML-RARα* negative patients is ultimately aimed at better evaluating the prognosis of such patients, so as to guide the selection and adjustment of clinical treatment strategies to prolong the survival time of patients as much as possible. The results of this study found that compared with the positive group of *PML-RARα* gene, the efficacy and prognosis of *PML-RARα* gene negative patients were poor, but the correlation between various examination indicators and the prognosis of this disease needs to be further explored and confirmed with more samples.

## Author contributions

**Conceptualization:** Bin Zhang.

**Formal analysis:** Diyuan Guo.

**Funding acquisition:** Bin Zhang.

**Writing – original draft:** Xinran Cao.
