# Comparison of laboratory characteristics and clinical prognosis of APL with negative and positive PML-RARα gene: Erratum

**DOI:** 10.1097/MD.0000000000040944

**Published:** 2024-12-06

In the article “Comparison of laboratory characteristics and clinical prognosis of APL with negative and positive PML-RARα gene”,^[1]^ which appears in Volume 103, Issue 47 of *Medicine*, Figure 1 and Tables 1, 2, and 5 were not correct. The correct versions appear here:

**Table 1:** Basic data of 14 PML-RARα-negative cases

No.	Sex	Age	Bone marrow promyelocytic ratio(%)	Flow cytometric immunophenotype
1	Female	66	19	CD11b,CD13,CD15,CD33,CD38,CD64,CD117,MPO
2	Female	50	95	CD8,CD11b,CD13,CD19,CD25,CD34
3	Female	59	30.5	CD13,CD33,CD38,CD64,CD117,MPO
4	Male	61	23	CD11b,CD13,CD15,CD33,CD64,CD117,MPO
5	Female	60	16.5	CD9,CD13,CD25,CD33,CD34,CD38,CD71,CD117
6	Female	31	73	CD9,CD15,CD33,CD38,CD64,CD117,HLA-DR
7	Female	16	63	CD9,CD13,CD33,CD38,CD117,MPO
8	Male	77	46	CD13,CD15,CD34,CD38,CD56,CD71,CD117,MPO
9	Male	56	67.5	CD11b,CD13,CD15,CD25,CD33,CD34,CD38,CD56,CD64,CD117, MPO
10	Male	43	20.5	CD33,CD38,CD64,CD117,MPO
11	Male	66	35	CD15,CD33,CD38,CD117,MPO
12	Female	18	67.5	CD4,CD11b,CD13,CD15,CD33,CD38,CD56,CD64,CD117,MPO,HLA -DR
13	Female	58	67	CD15,CD33,CD38,CD56,CD117,MPO
14	Female	50	93	CD4,CD13,CD15,CD33,CD38,CD56,CD64,MPO,HLA-DR

No.	Chromosome karyotype		Gene	First treatment plan and prognosis
1	46,XX[20]		PML-RARα(–)	ATRA×10d, died of cerebral hemorrhage
2	46,XY[19]		PML-RARα(–)	(ATRA+DA)×7d didn’t achieve CR, achieved CR after the second course of DA, and relapsed after the third course of DA
3	46,XX[11]		HOX11	DA×7d achieved CR, relapsed after 18 months
4	46,XY[4]		PML-RARα(–)	DA×7d achieved CR, relapsed after 8 months
5	46,XX[13]		PML-RARα(–)	DA×7d achieved CR, abandoned treatment in critical condition after CAG×14d
6	46,XX,t(6;11)(q27;q23) [3]/ 46,XX[17]		MLL- AF6	ATRA×5d, IA×7d achieved CR
7	46,XX[20]		PML-RARα(–)	IA×7d didn’t achieve CR
8	46,XY[20]		PML-RARα(–)	DA×7d didn’t achieve CR
9	44,X,-Y,del(5)(q13),der(15), -16, -21,+mar[20]		PML-RARα(–)	DA×7d didn’t achieve CR, died after (decitabine+CAG)×14d
10	46,XX[20]		PML-RARα(–)	ATRA×5d+TA×7d achieved CR
11	46,XY[19]		PML-RARα(–)	CAG×14d achieved CR
12	46,XX[16]		PML-RARα(–)	(ATRA+DA)×7d achieved CR, relapsed after 8 months
13	46,XX[20]		PML-RARα(–)	ATRA×16d+CAG×7d achieved CR
14	47,X,(X;10)(p12;p11),+8[11]/48,idem,+21[7]/46,XX[2]		PML-RARα(–)	CAG×14d achieved CR, relapsed after 7 months

CAG = ACLA + Ara-C + G-CSF, DA = DNR + Ara-C, IA = IDA + Ara-C, PML-RARα = promyelocytic leukemia-retinoic acid receptor-α, TA = THP + Ara-C.

**Table 2:** Comparison of peripheral blood routine, coagulation related indexes and bone marrow cell morphology between PML-RARα negative group and positive group

		PML-RARa negative group (n = 14)	PML-RARa positive group (n = 67)	P
Male/Female		5/9	29/38	0.602
Age(years)		50.79±18.03	47.94±15.73	0.590
Peripheral blood routine test				
WBC(×10^9^·L^-1^)		13.43(2.76,52.06)	2.02(1.25,5.82)	0.005
Hb(g·L^-1^)		80.64±18.58	80.18±12.31	0.942
PLT(×10^9^·L^-1^)		43.21±13.34	31(15,48)	0.532
Coagulation related indicators				
PT(s)		12.74±1.27	13.3(12.0,16.3)	0.177
APTT(s)		27.18±5.47	26.4±3.89	0.676
FIB(g·L ^-1^)		2.74(2.48,5.02)	1.66(1.02,2.95)	0.005
DD(mg·L ^-1^)		8.16(2.10,26.11)	9.95(3.56,27.63)	0.764
Bone marrow cell morphology				
Bone marrow promyelocytic ratio(%)		53.68±16.20	87.5±12.90	0.000
Auer body positive rate(%)		21.43	88.06	0.000
POX staining reaction degree	Strong positive(%)	42.86	80.59	0.007
	Positive(%)	28.57	10.45	0.091
	Weak positive(%)	28.57	8.96	0.065
	negative(%)	0	0	-

APTT = activated partial thromboplastin time, DD = D-dimer, FIB = fibrinogen, Hb = hemoglobin, PLT = platelet, PML-RARα = promyelocytic leukemia-retinoic acid receptor-α, POX = peroxidase, PT = prothrombin time, WBC = white blood cell.

**Table 5:** 15 cases of abnormal karyotype in PML-RARα positive group

No.	Chromosome karyotype
1	46,XY,t(15;17)(q22;q21)[7]/47,idem,+21[8]/46,XY[1]
2	46,XY,add(11)(q23),t(15;17)(q22;q21)[2]/46,XY[6]
3	46,XX,der(15)t(15;17)(q22;q21),der(17)add(17)(p12)t(15;17)[20]
4	46,XX,t(15;17)(q22;q21)[16]/47,idem,+mar[2]/46,XX[2]
5	47,XX,+8,der(15)t(15;17)(q22;q21),ider(17)(q10)t(15;17)[13]/47,t(15;17)(q22;q21), +mar[5]/46,XX[2]
6	46,XY,der(15)t(15;17)(q22;q21),ider(17)(q10)t(15;17)[12]/46,XY,t(15;17)(q22;q21) [4]/46,XY[4]
7	46,XX,del(7)(q11q22),t(15;17)(q22;q21)[12]/46,XX
8	46,XX,der(1)(p36)del(1)(q22q24),add(3)(p25),add(4)(q28),del(7)(q32),del(11)(p11), add(16)(q22)[8]/47,idem,+8[12]
9	46,XY,del(11)(p14),t(15;17)(q22;q21)[17]/46,XY[3]
10	46,XX,t(6;21)(q11;q21),t(15;17)(q22;q21)[19]/46,XX[1]
11	47,XX,del(7)(q12q22),t(15;17)(q22;q21),+mar[7]/46,XX[13]
12	45,XY,-6,add(7)(q36),t(15;17)(q22;q21),t(16;18)(q12;p11),add(19)(q12)[9]/46,idem, +mar[11]
13	46,XY,der(15)t(15;17)(q22;q21),ider(17)(q10)t(15;17)[17]/46,XY[3]
14	46,XY,t(12;19;17;15)(q13;q12;q21;q24)[11]/46,XY[9]
15	46,XX,der(15)t(15;17),ider(17q)t(15;17)

PML-RARα = promyelocytic leukemia-retinoic acid receptor-α.

**Figure 1.**
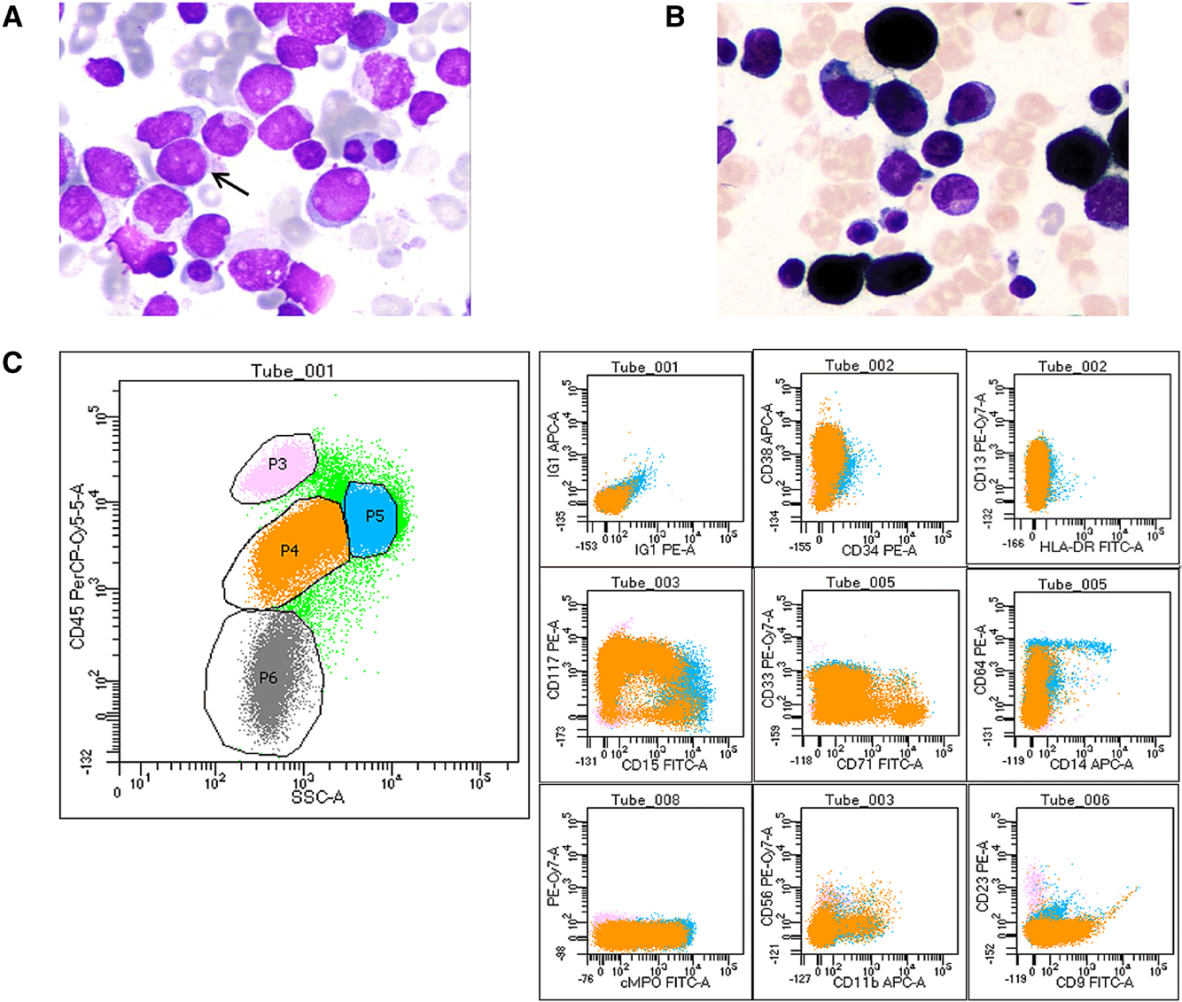
Morphology and immunophenotype of PML-RARα negative. Abnormal promyelocytes (arrows indicated) with varying cell sizes and low cytoplasm content were found in bone marrow smear stained with Raysh-Giemsa (magnification x1000, panel A). Pseudopod-like processes, purple-red small azurin particles, folded nuclei, clear nucleoli, and no Auer bodies were seen. The staining intensity of POX was strongly positive (panel B). Immunological analysis using flow cytometry was performed on 100,000 cells from the patient’s bone marrow. In the two-dimensional scatterplot of SSC/CD45, 64.1% of the cells were P4 cells, and the fluorescence intensity of CD45 PerCP and SSC were moderately expressed (about 103) (panel C left). The immunophenotype of P4 cells was as follows: CD34-/CD38+, HLA-DR-/CD13+, CD15-/CD117+, CD71-/CD33+, CD14-/CD64-, cMPO+, CD11b-/CD56-, CD9+ (21.8%)/CD23- (panel C). Consistent with myeloid naive cell immunophenotype. Molecular genetic results: 46, XX [20]; PML-RARα (-).
